# Investigating Different Mechanisms of Action in Combination Therapy for Influenza

**DOI:** 10.3389/fphar.2018.01207

**Published:** 2018-10-23

**Authors:** Kelli Melville, Thalia Rodriguez, Hana M. Dobrovolny

**Affiliations:** ^1^Physics Department, East Carolina University, Greenville, NC, United States; ^2^Department of Physics and Astronomy, Texas Christian University, Fort Worth, TX, United States

**Keywords:** influenza, antiviral, mathematical modeling, combination therapy, mechanism of action

## Abstract

Combination therapy for influenza can have several benefits, from reducing the emergence of drug resistant virus strains to decreasing the cost of antivirals. However, there are currently only two classes of antivirals approved for use against influenza, limiting the possible combinations that can be considered for treatment. However, new antivirals are being developed that target different parts of the viral replication cycle, and their potential for use in combination therapy should be considered. The role of antiviral mechanism of action in the effectiveness of combination therapy has not yet been systematically investigated to determine whether certain antiviral mechanisms of action pair well in combination. Here, we use a mathematical model of influenza to model combination treatment with antivirals having different mechanisms of action to measure peak viral load, infection duration, and synergy of different drug combinations. We find that antivirals that lower the infection rate and antivirals that increase the duration of the eclipse phase perform poorly in combination with other antivirals.

## 1. Introduction

Influenza, more commonly known as the flu, is a seasonal illness that has symptoms of runny nose, cough, fever and an aching body (Khandaker et al., 2011). While most people recover fairly quickly, influenza can be lethal, especially in children and the elderly (Pop-Vicas and Gravenstein, 2011; Ruf and Knuf, 2014). There are vaccines to prevent influenza, though unfortunately, they are strain-specific (Jang and Seong, 2014), change annually, and don't always match the circulating strain (Dos Santos et al., 2016). This sometimes leaves antivirals as our only defense against influenza. However, the usefulness of antivirals is limited by the fast mutation rate of influenza (Sanjuán and Domingo-Calap, 2016; Villa and Lässig, 2017) and its ability to quickly develop resistance to antivirals (Perelson et al., 2012; Dobrovolny and Beauchemin, 2017). Of the two classes of drugs currently approved for use against influenza, one (adamantanes) is rarely used anymore due to high resistance in circulating strains (Bright et al., 2006; Dong et al., 2015). The class of drugs most broadly used against influenza is the neuraminidase inhibitors (NAIs), such as oseltamivir, zanamivir, and peramivir. While most strains of influenza are currently susceptible to NAIS, resistance to oseltamivir rose rapidly to 98–100% of circulating strains in the 2008–2009 flu season (Dharan et al., 2009; Zaraket et al., 2010), showing that resistance to these antivirals can suddenly emerge. A number of polymerase complex inhibitors (pimodivir, faviparivir, and S-033188) are also in development (Shaw, 2017; Yuan et al., 2018). Since these antivirals are fairly new, little is yet known about influenza's ability to develop resistance, although many think that since these domains are highly conserved, resistance is less likely to develop (Zhang et al., 2018). As new antivirals are developed (Naesens et al., 2016), physicians will need to develop treatment strategies that will limit the emergence of drug resistance.

Combination therapy, the use of two or more antivirals, is one possible strategy for limiting drug resistance. While influenza can very quickly develop resistance to a single antiviral, it takes longer to develop resistance to two drugs simultaneously (Perelson et al., 2012). In using two drugs then, we ensure that nearly all virus produced during the infection are susceptible to at least one of the drugs, so they will not replicate and the infection will be suppressed. There have been many experimental studies, both *in vivo* and *in vitro*, investigating the effect of different combinations of influenza antivirals (Hayden et al., 1980; Smee et al., 2002, 2009, 2010a,b; Govorkova et al., 2004; Ilyushina et al., 2006, 2007, 2008; Masihi et al., 2007; Bantia et al., 2010; Duval et al., 2010; Kim et al., 2010; Chen et al., 2011; Haasbach et al., 2013; Seo et al., 2013; Tarbet et al., 2014; Belardo et al., 2015; Morokutti-Kurz et al., 2015; Marathe et al., 2016; Beigel et al., 2017; de Mello et al., 2018), with some examining not just combinations of two drugs, but even examining triple combinations (Nguyen et al., 2009, 2010, 2012; Hoopes et al., 2011; Kim et al., 2011; Lu et al., 2015; Pavlova et al., 2018). These experimental studies are largely limited to combinations of antivirals from the two widely available classes of influenza antivirals, neuraminidase inhibitors (NAIs) and adamantanes.

The mechanisms of action of NAIs and adamantanes are at different points in the viral replication cycle with adamantanes acting to block viral replication (Abed et al., 2005) and NAIs acting to block viral release (Gubareva et al., 2000; Abed et al., 2002). However, new antivirals with different mechanisms of action are being developed (Koszalka et al., 2017; White et al., 2018; Zabrodskaya et al., 2018), and will potentially be used in combination therapy (Loregian et al., 2014; Koszalka et al., 2017). With the development of these new antivirals, there has been some interest in exploring how the mechanism of action of antivirals involved in combination therapy affects the effectiveness of combination therapy (Dunning et al., 2014; Popov et al., 2018). This is most often measured by assessing, via *in vitro* experiments, the synergy or antagony of the drug combination. Due to interactions between the drugs, the effect of their combination can be larger (synergy) or smaller (antagony) than the sum of their individual effects (Bliss, 1939; Loewe, 1953; Berenbaum, 1989). Unfortunately, experimental examination of a wide range of combination therapy doses is costly and time-consuming.

Mathematical models can help in the effort to find optimal combination therapy doses. Within host mathematical models of influenza have previously been used to study many aspects of antiviral treatment including extracting of drug efficacy parameters (Beauchemin et al., 2008; Brown et al., 2011; Beggs and Dobrovolny, 2015; Liao et al., 2017), treatment of severe influenza (Dobrovolny et al., 2010, 2011; Deecke and Dobrovolny, 2018), emergence of drug resistance (Handel et al., 2007; Perelson et al., 2012; Hur et al., 2013; Canini et al., 2014; Dobrovolny and Beauchemin, 2017; Deecke and Dobrovolny, 2018), and to optimize antiviral treatments (Perelson et al., 2012; Heldt et al., 2013; Hur et al., 2013; Canini et al., 2014). While there are some mathematical models that attempt to model infections in patients by including an immune response (Dobrovolny et al., 2013; Cao and McCaw, 2015; Cao et al., 2015; Price et al., 2015; Zarnitsyna et al., 2016; Yan et al., 2017), the lack of appropriate human data for parameterizing and validating these models limits their predictive ability (Dobrovolny et al., 2013; Boianelli et al., 2015). However, simpler mathematical models can successfully reproduce *in vitro* dynamics (Beauchemin and Handel, 2011; Pinilla et al., 2012), and since mathematical models can quickly and efficiently simulate hundreds of combinations of doses, they are ideally suited as preliminary studies to ascertain whether combination therapy is effective and, if so, which combinations of doses produce the best results.

In this paper, we use an *in vitro* mathematical model of influenza infection to study combination therapy of influenza antivirals with different mechanisms of action. We measure the peak viral load, infection duration, and synergy/antagony of the various drug combinations to determine whether mechanisms of action pair better in combination therapy. We find that drugs that lengthen the eclipse phase and drugs that decrease the infection rate perform poorly, using all three measures, in combination with all other drugs.

## 2. Materials and methods

### 2.1. Model

We use a simple model of viral infection (Pinilla et al., 2012) given by the equations

(1) $$\begin{array}{r} \begin{array}{cc} Ṫ & =-\beta TV\\ {Ė}_{1} & =\beta TV-\frac{n_{E}}{\tau_{E}}E_{1}\\ {Ė}_{j} & =\frac{n_{E}}{\tau_{E}}E_{j-1}-\frac{n_{E}}{\tau_{E}}E_{j}\text{for}j=2,\ldots ,n_{E}\\ {İ}_{1} & =\frac{n_{E}}{\tau_{E}}E_{n_{E}}-\frac{n_{I}}{\tau_{I}}I_{1}\\ {İ}_{j} & =\frac{n_{I}}{\tau_{I}}I_{j-1}-\frac{n_{I}}{\tau_{I}}I_{j}\text{for}j=2,\ldots ,n_{I}\\ \overset{{}^{\circ}}{V} & =p\overset{n_{I}}{\underset{j=1}{\sum }}I_{j}-cV. \end{array} \end{array}$$

In this model, virus *V* infects healthy target cells *T* at a rate β. Once infected, the cells move into the eclipse phase *E*_*j*_ during which there is internal replication of viral proteins and RNA, but no external production of virus. After some average time τ_*E*_, the cells move into the infectious phase *I*_*j*_ where they are actively producing virus at rate *p*. Virus is cleared from the system at a clearance rate *c*. After an average time τ_*I*_, infectious cells die. Both the eclipse and infectious phases are modeled as having Erlang distributions represented as the multiple (*n*_*E*_ eclipse and *n*_*I*_ infectious) compartments in each phase. Recent work has suggested that this distribution more faithfully reproduces all aspects of viral dynamics (Holder and Beauchemin, 2011; Kakizoe et al., 2015; Beauchemin et al., 2017).

In order to perform simulations with our model, we need estimates of the parameter values. We used parameter values determined by Pinilla et al. (2012) through fits of this model to data from *in vitro* infections of influenza A/Québec/144147/09 in MDCK cells. Parameter values are given in Table 1.

**Table 1:** Parameter values used for simulations.

**Parameter**	**Meaning**	**Value**
*T*_0_	Initial number of target cells	10^6^ cells
*c*	Viral clearance rate	0.13 /h
τ_*E*_	Duration of the eclipse phase	6.6 h
τ_*I*_	Infectious lifespan	49 h
*n*_*E*_	Number of eclipse compartments	30
*n*_*I*_	Number of infectious compartments	100
β	Infection rate	4.260 × 10^−4^ (h · TCID_50_)^−1^
*p*	Viral production rate	176 TCID_50_ · (h · cell)^−1^

*Values are taken from Pinilla et al. (2012)*.

### 2.2. Modeling the drug effect

To model the effectiveness of influenza antivirals, we use the efficacy, ε, a parameter that varies from 0 to 1. An efficacy of 0 means the drug has no effect while an efficacy of 1 means the drug is completely effective. The efficacy of a drug is related to the drug dose through the *E*_max_ model (Holford and Sheiner, 1981),

(2) $$\begin{array}{r} \varepsilon =\frac{\varepsilon_{\text{max}}\cdot D^{\gamma }}{D^{\gamma }+\text{I}{\text{C}}_{50}^{\gamma }}, \end{array}$$

where *D* is the dose of the antiviral, ε_max_ is the maximum possible drug efficacy, IC_50_ is the drug dose needed to achieve half the maximum effect, and γ is the Hill coefficient. Biologically, γ is determined by the number of binding reactions needed for the drug to function (Weiss, 1997), which is assumed to be 1 for influenza antivirals (Beauchemin et al., 2008; Beggs and Dobrovolny, 2015). We also assume that ε_max_ =1 so that we can explore the full range of possible behavior. Finally, we set IC_50_ =1 which amounts to expressing drug doses relative to the IC_50_, i.e., *D* → *D*/IC_50_.

In this study, we are interested in examining how antiviral mechanism of action affects the efficacy of combination therapy of influenza. Therefore, we examine not only currently available antivirals, but also hypothetical antivirals with other mechanisms of action. We model different mechanisms of action by applying drug efficacy to different parameters of the model. It is important to note that the model is quite general in that most biological processes are not explicitly represented. This means that it is not always clear which processes are captured by each parameter and some processes may affect more than one parameter. For example, the boundary between cell infection, represented by β, and the intracellular processes that are part of the eclipse phase, represented by τ_*E*_, is not well-defined; fusion and endocytosis fall into this fuzzy boundary region and might well affect both model parameters.

The mechanisms of action for antiviral drugs that we modeled are:

- **Reducing infection rate**. This is modeled by applying the efficacy to the parameter β → (1−ε)β for β in both the target cell and eclipse cell equations. This represents an antiviral that blocks entry into the cell.
- **Protecting target cells**. To do this we place the efficacy on the parameter β → (1 − ε)β, but only for the β that appears in the eclipse equation. This represents an antiviral that blocks intracellular processes, but not entry into the cell. Cells containing virus are removed from the target cell class, but cannot be infected and so can be thought of as being “protected” from infection. This was shown to be the best mathematical model for replicating the effects of the influenza antiviral amantadine (Beauchemin et al., 2008). This mechanism will be denoted by β_2_.
- **Reducing virion production rate**. For this mechanism, we put the efficacy on *p* → (1 − ε)*p*. This is often used to model the action of neuraminidase inhibitors (Baccam et al., 2006; Handel et al., 2007; Dobrovolny et al., 2011).
- **Increasing the rate of viral clearance**. In this case, the efficacy is applied to *c* → *c*/(1 − ε). In patients, this most likely represents a drug that stimulates the adaptive immune response, particularly antibodies, to enhance clearance of the virus (Taylor and Dimmock, 1985a,b), but could also represent a drug that inactivates virus (Fujimori et al., 2012).
- **Increasing the length of the eclipse phase**. A drug effect on parameter τ_*E*_ → τ_*E*_/(1 − ε) represents an antiviral that delays assembly of the virions. There could be several possible mechanisms for this (Heldt et al., 2013), such as delay of production of proteins or RNA.
- **Decreasing the lifespan of infectious cells**. A drug effect on parameter τ_*I*_ → (1 − ε)τ_*I*_ could also represent a stimulant of the immune response in patients, although in this case increasing the cytotoxic T lymphocytes which are responsible for killing infected cells (Zweerink et al., 1977; Mbawuike et al., 2007), but could also represent a drug that stimulates autophagy of infected cells (Feizi et al., 2017).

### 2.3. Measuring the effect of combination therapy

We use our model to determine which combination of antivirals are the best at treating influenza. We use two measurements of the viral titer to assess the efficacy of treatment, the maximum viral titer *V*_max_ and infection duration, *T*_inf_. The maximum viral titer is indicative of the viral burden in patients and is thought to be a measure of the transmissibility of the infection (Handel et al., 2009). The infection duration is defined as the time the viral titer remains above $10^{4}\text{TCI}{\text{D}}_{50}$, as described in Dobrovolny et al. (2010).

A quantity that is often used to characterize combination therapy is synergy or antagony. If the effect of a combination of antivirals is better than expected based on the individual effects, the combination is said to be synergistic; if the effect of the combination is worse than expected, the combination is said to be antagonistic. If we assume a multiplicative effect for drugs, known as Bliss synergy (Bliss, 1939), this is calculated via

(3) $$\begin{array}{r} S=W-\left[M+\left(1-M\right)N\right], \end{array}$$

where *W* is the observed percent inhibition of viral titer due to the combination of two drugs, and *M* and *N* are the observed percent inhibition of each drug individually (Koizumi and Iwami, 2014). A positive value of *S* represents a synergistic combination, and a negative value represents antagony.

An alternative assumption, known as Loewe additivity (Loewe, 1953), assumes that the effect of drug combinations is additive. Synergy in this case is measured using the combination index (CI)

(4) $$\begin{array}{r} \text{CI}=\frac{D_{1}^{*}}{D_{1}}+\frac{D_{2}^{*}}{D_{2}}, \end{array}$$

where *D*_1_ and *D*_2_ are the doses of the two drugs that result in a particular effect during monotherapy, and $D_{1}^{*}$ and $D_{2}^{*}$ are the doses of the two drugs in combination that result in the same effect. If the CI is greater than 1, the drugs are considered synergistic, while if the CI is less than 1, the drugs are considered antagonistic. Note that for these calculations, viral titer is measured at a specific time; we used 72 h, a time that is commonly used in experimental measurements of synergy (Smee et al., 2002, 2009; Ilyushina et al., 2006, 2008; Nguyen et al., 2009, 2010).

## 3. Results

### 3.1. Curing the infection

For ordinary differential equation models, whether or not an infection progresses is determined by the basic reproductive number, *R*_0_, which is defined as the number of secondary infections produced by a single infected cell in a heterogeneous population. *R*_0_ for this model is given by

(5) $$\begin{array}{r} R_{0}=\frac{p\beta \tau_{I}T_{0}}{c}. \end{array}$$

If *R*_0_ > 1, we will have an infection and if *R*_0_ < 1, infection is suppressed. Modeling the application of drug as described in the Methods section results in modification of *R*_0_, such that *R*_0, treated_ = (1 − ε_1_)(1 − ε_2_)*R*_0_, where ε_*i*_ are the efficacies of each of the two drugs being used in combination. Applying the condition that *R*_0, treated_ = 1, and keeping the assumption that the Hill coefficient is 1 (γ=1), we can find the boundary between combinations of doses that prevent infection and those that don't.

(6) $$\begin{array}{r} D^{\left(i\right)}=\text{I}{\text{C}}_{50}^{\left(i\right)}\frac{1-R_{0}f^{\left(j\right)}}{R_{0}f^{\left(j\right)}\left(1-\varepsilon_{\text{max}}^{\left(i\right)}\right)-1}, \end{array}$$

where *i, j* = 1, 2 indicating each of the two drugs in the combination, and *f*^(*i*)^ is given by

(7) $$\begin{array}{r} f^{\left(i\right)}=\frac{\text{I}{\text{C}}_{50}^{\left(i\right)}+D^{\left(i\right)}\left(1-\varepsilon_{\text{max}}^{\left(i\right)}\right)}{D^{\left(i\right)}+\text{I}{\text{C}}_{50}^{\left(i\right)}}. \end{array}$$

Using Equation (2), with our assumptions of ε_max_=1 and IC_50_=1, to write the condition in terms of drug dose, we have

(8) $$\begin{array}{r} D^{\left(2\right)}=\frac{R_{0}}{1+D^{\left(1\right)}}-1. \end{array}$$

Note that this analysis does not capture the effect of a drug effect applied to τ_*E*_ since τ_*E*_ does not appear in *R*_0_.

**Figure 1:**
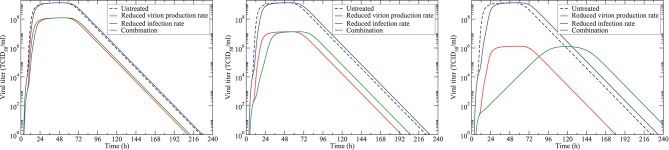
Model simulations of antiviral treatment. The untreated infection is given by the black dashed line. Monotherapy with an antiviral reducing virion production is shown in red. Monotherapy with an antiviral reducing the infection rate is shown in blue. The combination of the two (at the same doses) is shown in green. The left panel shows infections when the antiviral is treated with doses of 10 × IC_50_; the center panel shows doses of 100 × IC_50_; and the right panel shows doses of 1,000 × IC_50_.

The mathematical model (Equation 1) can be computationally solved, giving a picture of how viral load changes as different drugs are applied. In Figure 1, we show the model predictions of treatment with an antiviral that reduces virion production rate (red line), an antiviral that reduces infection rate (blue line), and their combination (green line). The three panels show antiviral doses of 10 × IC_50_ (left), 100 × IC_50_ (center), and 1,000 × IC_50_. We see that a drug that reduces infection has little effect on the viral titer, shifting the curve slightly to the right at high doses, but not affecting the peak viral load much. The drug that reduces virion production has a larger effect. At a dose of 10 × IC_50_, the combination regimen is no different than monotherapy with a drug that reduces production of virions. At higher doses, the combination treatment reflects the reduced peak viral load of the drug reducing virion production and the rightward shift of a drug that reduces infection rate.

**Figure 2:**
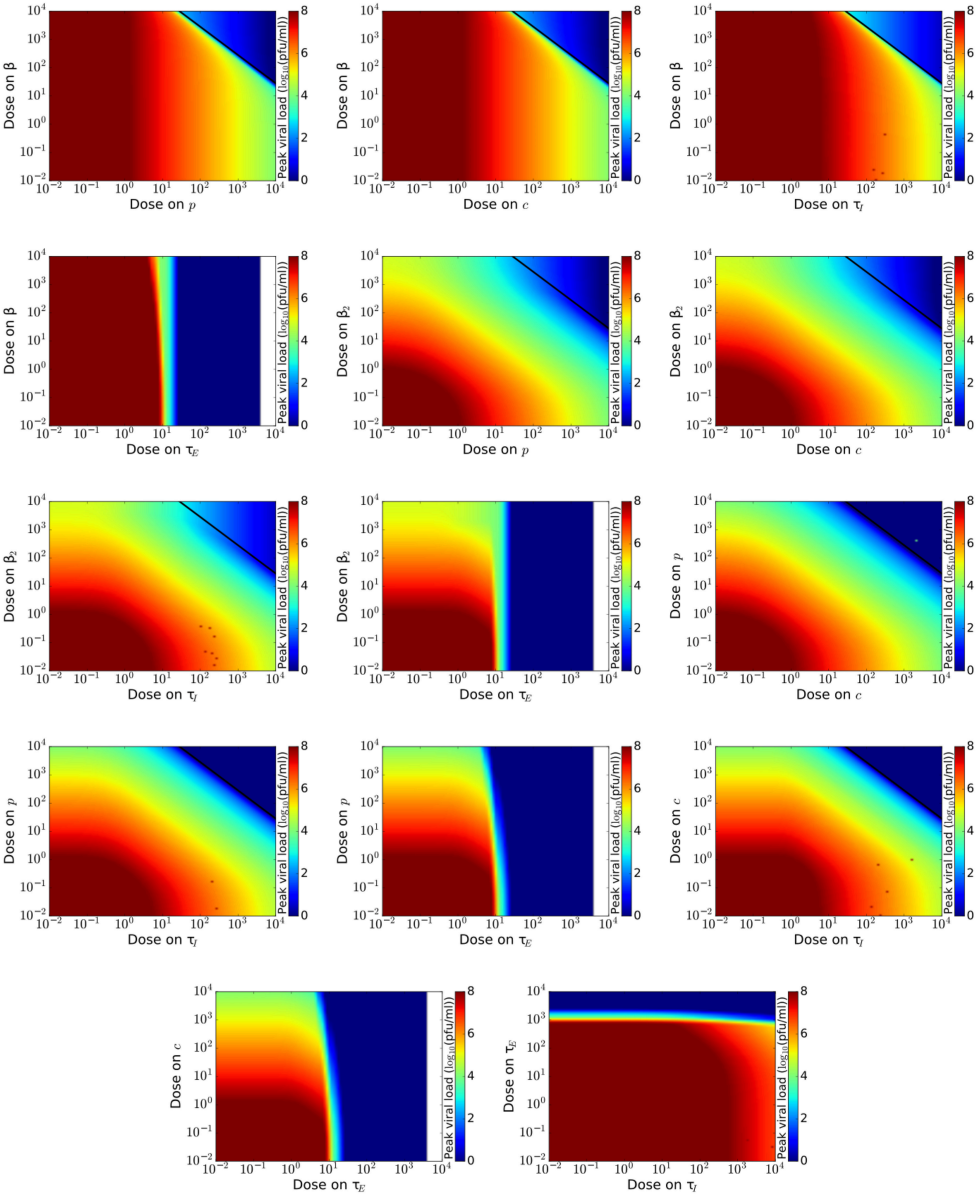
Maximum viral titer for a range of doses of various combinations of influenza antivirals. Lowest peak viral titer is in blue with highest peak values indicated in red. The black line indicates the theoretical curve given by Equation (8) that delineates regions where the infection is cured.

**Figure 3:**
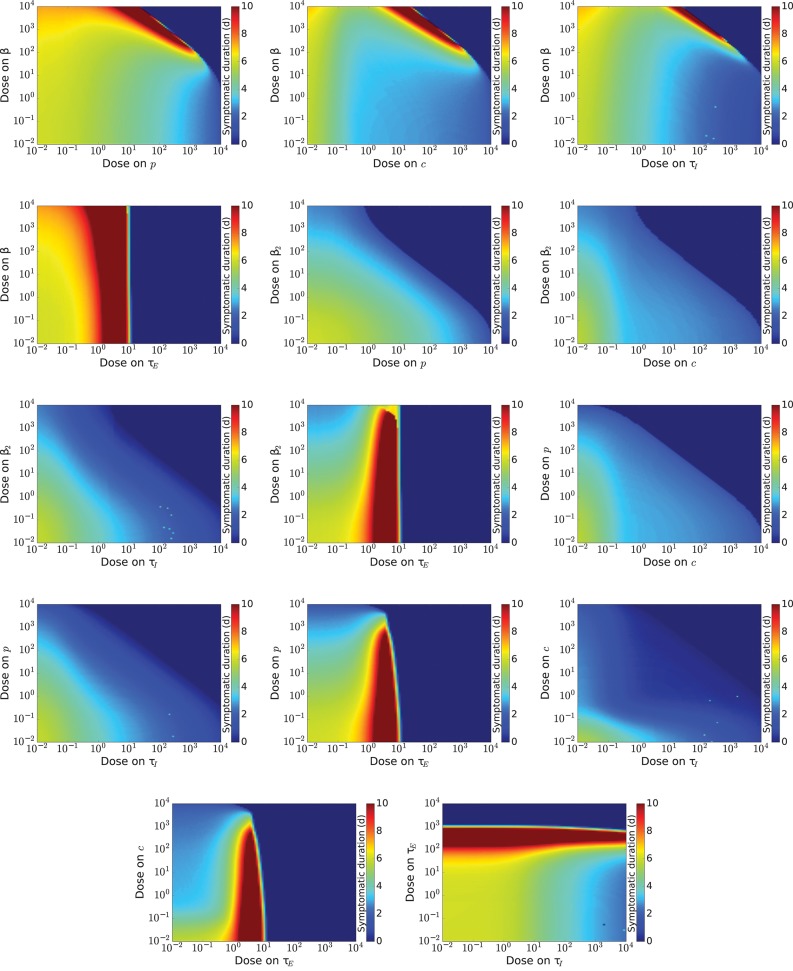
Infection duration for a range of doses of various combinations of influenza antivirals. Regions where the infection has been cured, (duration=0) are indicated by dark blue, while regions where the infection is longer than 10 days are indicated by red.

To get a more complete view of drug effect, we investigate the effect of combination therapy on measures of disease burden, *V*_max_ and *T*_inf_. We examine possible combinations of theoretical antivirals (14 pairs), excluding combinations of drugs modeled as acting on β and β_2_. For each dose combination, we simulate an infection and measure *V*_max_ and *T*_inf_. Results for *V*_max_ are shown in Figure 2 and results for *T*_inf_ are shown in Figure 3. The boundary between infection and no infection, given by Equation (8), is indicated by the black line on graphs in Figure 2. Note that while we investigate the effect of antivirals up to doses 10${}^{4}\text{I}{\text{C}}_{50}$, such high doses are not achievable for many antivirals due to toxicity.

While the boundary between infection and no infection is the same for most drug combinations, we can see from Figure 2 that different mechanisms of action of the antivirals affect how quickly viral load is reduced as we approach the boundary. For example, a drug that reduces infection rate (reducing β) does not assist in lowering viral load no matter which other drug is used in combination. This is seen in the first four graphs of Figure 2 which show no change in viral load as dose on β (y-axis) is varied. We can also see that drugs that increase the duration of the eclipse phase appear to reduce the viral load to zero beyond a certain dose (about 10 IC_50_), although this is just a computational effect. A drug that increases the duration of the eclipse phase simply delays the time of the peak viral titer (González-Parra and Dobrovolny, 2018), in this case pushing the peak beyond the duration of the simulation. An infection that grows that slowly, however, is likely to be suppressed by the patient's immune response (Beauchemin and Handel, 2011). The remaining drug combinations, those not including a drug reducing infection rate or a drug increasing eclipse duration, all have a similar effect on peak viral load.

Since the effect of different drug combinations on viral load is so similar, we look for other measures of infection severity that might help differentiate certain drug combinations. The infection duration is shown in Figure 3. We again see boundaries, for all drug combinations, beyond which *T*_inf_ falls to 0 and there is no infection. For combinations that include a drug that reduces infection rate, *T*_inf_ increases quite drastically if the dose of either drug is just below this boundary. We see a similar increasing *T*_inf_ when a drug that increases the eclipse phase is part of the combination due to the delay of the replication cycle as the eclipse phase increases. The remaining drug combinations show gradual decrease of *T*_inf_ as drug dose of either drug in the combination is increased. We can, however, see some slight differences in how quickly *T*_inf_ decreases. We find that the combination of a drug increasing the clearance rate and a drug decreasing the infectious lifespan reduces *T*_inf_ is the most effective at reducing the duration of the infection since it requires lower doses than the other combinations to achieve short infection durations.

### 3.2. Synergy

Of particular interest is whether a drug combination improves outcomes over treatment with a single drug. There are two models for drug interaction; one assumes a multiplicative effect (Bliss synergy) (Bliss, 1939), and one assumes an additive effect (Loewe, 1953). Since we are not investigating specific antivirals, we cannot make assumptions about how the antivirals might interact, so we include information about both types of synergy here.

**Figure 4:**
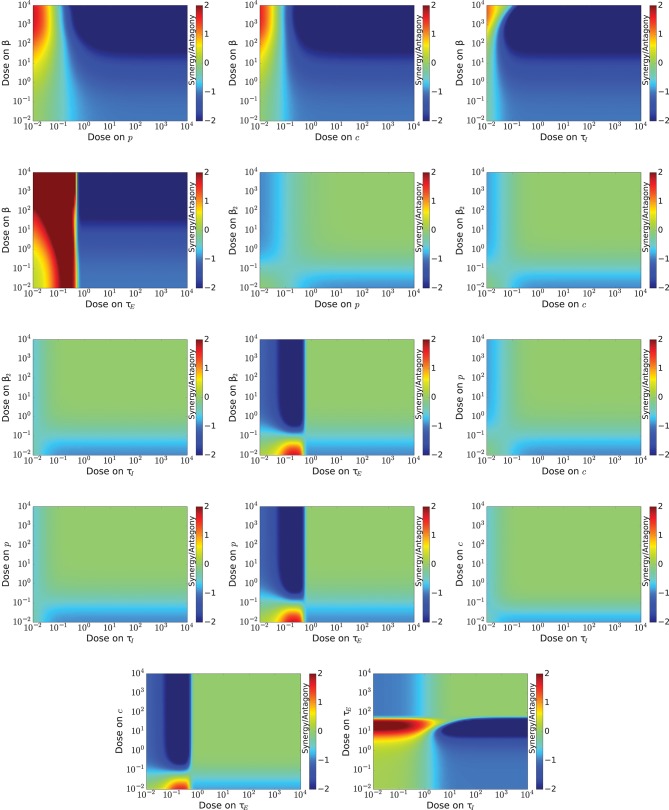
Synergy for a range of doses of various combinations of influenza antivirals. Antagony of the antivirals is indicated by regions of blue, while regions of synergy are indicated by red. Note that most combinations result in neither synergy nor antagony (pale green).

The Bliss synergy/antagony of the different drug combinations is shown in Figure 4. In these figures, blue indicates antagony, red indicates synergy, and green indicates no enhancement or impediment of the antiviral effect. Drug combinations that include an antiviral that decreases infection rate show antagony over a broad range of doses. Drug combinations that include a drug that increases the eclipse duration also have large regions of antagony. The remaining drug combinations are largely neutral, showing little synergy or antagony over most combinations of doses. Particularly interesting is the combination of an antiviral that reduces infection rate and one that prolongs the eclipse phase (second row, left panel) where there is high synergy at low doses of the drug that prolongs the eclipse phase and antagony at high doses of this antiviral. Remember that synergy is a measure of whether the drug combination had a larger effect than expected. At low doses, the drugs are not expected to have much effect, so as long as the combination outperforms that low expectation, synergy will be high. Both of these antivirals shift the viral titer curve to the right. On their own, they will not shift the curve much (even up to very high doses for a drug that reduces infection rate, as seen in Figure 1), but combined they shift the curve just enough to have the peak move past the 72 h measurement time resulting in a large drop in the viral titer measurement.

**Figure 5:**
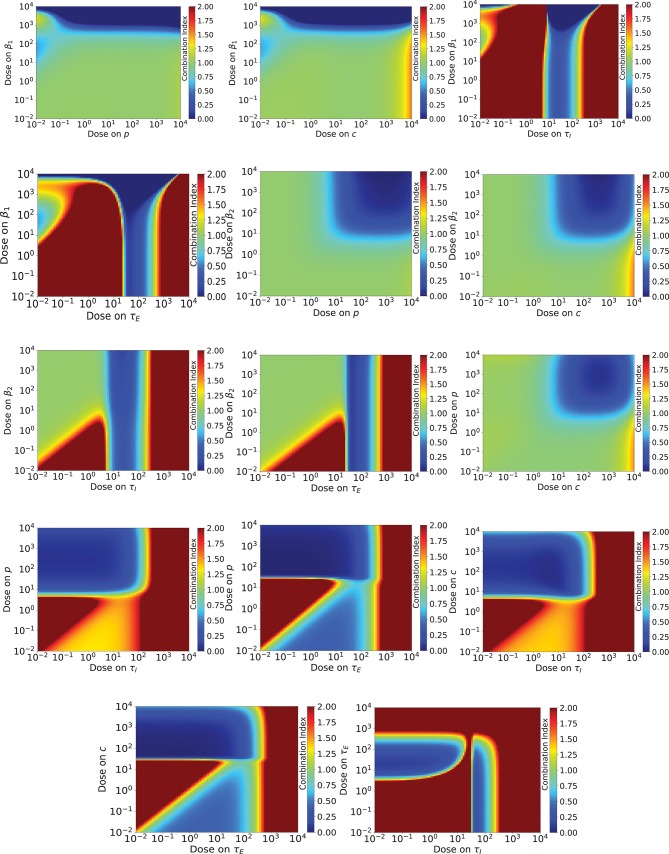
Combination index for a range of doses of various combinations of influenza antivirals. Antagony of the antivirals is indicated by regions of blue, while regions of synergy are indicated by red. A combination index of one (no effect) is pale green.

The combination index of the different drug combinations is shown in Figure 5. Like the previous figure, blue indicates antagony, while red indicates synergy. In this case, no effect is given by CI = 1. If we assume Loewe additivity for our antivirals, we see many more regions of synergy than for Bliss synergy. There are only five drug combinations (β_1_/*p*, β_1_/*c*, β_2_/*p*, β_2_/*c*, *p*/*c*) where the majority of dose combinations show antagony or no effect. For the remaining drug combinations, the CI varies rapidly, going from anatagony to synergy over a very small range of doses.

## 4. Discussion

In this paper, we computationally evaluated combinations of theoretical influenza antivirals to assess the effect of antiviral mechanism of action on drug interactions. We found that combination therapies that include drugs that reduce the infection rate or combination therapies that include a drug that lengthens the eclipse phase show markedly different effects on peak viral load, infection duration, and synergy than other combination therapies. The decreased efficacy in reducing viral load, dose combinations that lead to increased infection duration, and large number of dose combinations that produce antagonistic reactions suggest that antivirals with these two mechanisms of action are not good candidates for combination therapy. Combination therapies using antivirals with other mechanisms of action have similar effects on peak viral load, infection duration, and synergy, although a combination of an antiviral that increases viral clearance and an antiviral that decreases infectious cell lifespan reduces infection duration at lower doses than other drug combinations, perhaps making it the best combination. Note that both of these antivirals decrease the duration of the viral decay phase of influenza. Since this phase is typically much longer than the viral growth phase (Smith et al., 2010; Beauchemin and Handel, 2011), a fractional reduction of the duration of this phase results in a greater shortening of the infection than a similar fractional reduction in the duration of the growth phase.

While we have identified a “best” antiviral combination by using infection duration, other measurements could lead to a different conclusion. Other features of the viral titer curve, besides peak viral load or some measure of infection duration, are sometimes used to assess the effectiveness of antiviral treatment (Beggs and Dobrovolny, 2015). These include the area under the viral titer curve (AUC) (Ryan et al., 1994; Hayden et al., 1999; Heldens and van den Hoven, 2002; Rayner et al., 2013; Beggs and Dobrovolny, 2015) or the viral growth and decay rates (Beggs and Dobrovolny, 2015). Measurement of these features might allow further differentiation of the role of antiviral mechanism of action in combination therapy, although the value of these measures in terms of clinical benefit to the patient are unclear. The most common method of assessing efficacy of an antiviral (or combination of antivirals) is to measure the viral load at a specific time (Noah et al., 2007), although the efficacy estimated in this manner is known to depend on the measurement time (Stresser et al., 2014).

Besides looking at features of the viral titer curve, other factors could play a role in deciding which antiviral combinations are most beneficial to patients. Combination therapy has been proposed as a strategy to decrease the occurrence of drug resistance (Dunning et al., 2014). A recent modeling study suggests that mechanism of action of an antiviral plays a role in how quickly drug resistant mutants emerge during an influenza infection (Dobrovolny and Beauchemin, 2017) during monotherapy. This effect could carry over to combination therapies such that certain antiviral combinations will be more effective in blocking the appearance of drug resistant mutants, a factor that should be considered when determining optimal drug combinations for treating influenza. The toxicity/side-effects of antivirals also needs to be considered when determining optimal treatment strategies. Other factors that could be considered when designing drug combinations are the cost or cost-benefit ratio of the medications (Burch et al., 2009) and toxicity or side-effects of the medications.

The mathematical model used in this study is fairly simple and does not include a full description of all biological processes involved in influenza replication. We do not include an explicit immune response since there is still no consensus on the correct mathematical formulation of immune responses (Dobrovolny et al., 2013), although this will be needed to properly assess combination therapy *in vivo*. A recent modeling study has indicated that inclusion of an immune response alters the predicted effect of antivirals (Cao and McCaw, 2015). Inclusion of an immune response would also allow for more accurate representation of immune-stimulating antivirals (Zweerink et al., 1977; Taylor and Dimmock, 1985a,b; Mbawuike et al., 2007). More broadly, due to the generality of the model, we also do not fully capture the full range of possible mechanisms of action of influenza antivirals (Heldt et al., 2013; Liao et al., 2017), although this could be corrected by using more detailed models if combination therapy of specific antivirals needs to be investigated. While this study might not be detailed enough to make predictions about specific antiviral combinations, the model used here is sufficient to show that antiviral mechanisms of action affect the effectiveness of combination therapy. Clinicians and drug developers should consider interactions between different mechanisms of action when developing combination therapies.

## Data availability statement

The datasets generated for this study can be found in github [https://github.com/hdobrovo/combination_therapy.git].

## Author contributions

HD conceived the experiments. KM and HD conducted the experiments. KM, TR, and HD analyzed the results, KM and HD wrote the manuscript. All authors reviewed and approved the manuscript.

### Conflict of interest statement

The authors declare that the research was conducted in the absence of any commercial or financial relationships that could be construed as a potential conflict of interest.
